# Provident Scheme for Sussex

**Published:** 1921-01-29

**Authors:** 


### PROVIDENT SCHEME FOR SUSSEX.

THE office of this scheme, full particulars of which we published last week (page 385), is at 4 St. George's Place, Brighton.

